# Carotenoid Markers of Dietary Exposure and Nutritional Status

**DOI:** 10.3390/nu15102359

**Published:** 2023-05-18

**Authors:** Begoña Olmedilla-Alonso

**Affiliations:** Department of Metabolism and Nutrition, Institute of Food Science, Technology and Nutrition (ICTAN-CSIC), C/José Antonio Novais, 6, 28040 Madrid, Spain; bolmedilla@ictan.csic.es; Tel.: +34-91-549-2300

Carotenoids are lipophilic isoprenoid compounds synthesized by photosynthetic organisms and some non-photosynthethic prokaryotes and fungi. They are widely present throughout nature and approximately one thousand have been described to date. As humans cannot synthesize carotenoids, they must be obtained from the diet. It is worth noting that, in a varied diet containing fruit and vegetables, approximately 50 carotenoids are available for absorption, but only 6 found in the human bloodstream have been studied in depth (β-carotene, α-carotene, β-cryptoxanthin, lutein, zeaxanthin, and lycopene), in order to verify their impact on human health/disease. Some carotenoids of undeniable nutritional importance are used as vitamin A precursors (β-carotene, α-carotene, β-cryptoxanthin, and other carotenoids scarcely found in our diets). Carotenoids also perform other biological activities that may confer their beneficial effects against some chronic diseases (i.e., lutein in the eye and the brain) [1]. Since the 1980s, the number of studies on the biological activity of carotenoids, in relation to human health promotion, has grown exponentially. These have focused on a wider variety of carotenoids, including astaxanthin, neoxantin, violaxanthin, phytoene, and phytofluene, among others [1,2]. 

However, concerning the relationship between the dietary intake of carotenoids and their concentrations in tissue and human health, both positive and negative effects/correlations have been described in the literature [1,3]. Therefore, before recommendations can be issued, more data from human studies, including dietary intake and nutritional status data on as many carotenoids as possible in well-defined groups, are needed. Thus, the purpose of this Special Issue is to collect the recently reported evidence that enhances our knowledge of the intake and status of carotenoid markers and the methodological and other factors relevant to assessing and interpreting these carotenoid markers of dietary intake/exposure and nutritional status in human health and disease. This Special Issue includes five papers, two of which focus on dietary assessments: one on phytoene and phytofluene intake in the Spanish population [4] and the other on a dietary assessment method, using the Diet ID^TM^ to estimate carotenoid intake [5]. Regarding the other three papers: one is a review of the registered clinical trials on carotenoids (food and/or food supplements) to study their health effects [6], while the remaining two report on the results of a lutein-rich fruit or vegetables intervention study on lutein status markers [7] and phytoene and phytofluene dietary intake and status markers [8].

When assessing the dietary intake of carotenoids, it is important to evaluate food consumption, which can be studied using a wide variety of methods, and to access food composition tables containing individual carotenoid data. Food intake can be assessed in different ways, each featuring strengths and weaknesses [9]. To mitigate the latter, new assessment methods are frequently being proposed, because this is a key step when studying the correlation between intake and status. In this Issue, a rapid assessment method using the application Diet ID^TM^, which employs an image-based algorithm to identify dietary patterns, is proposed to estimate the nutrient intake in general, as well as the carotenoid intake [5]. Carotenoid intake assessed using this method is correlated with skin carotenoid scores and plasma carotenoids. Although the sample is small, it appears to be a promising tool for nutrition research. 

It should also be noted that food composition tables/databases rarely show individual carotenoid data and that the important issue of bioavailability is missing as well [2]. This appears to be crucial information, as different carotenoids and carotenoid forms exhibit different bioavailabilities (i.e., free vs. ester forms and xanthophylls vs. carotenes) [10,11]. Also worth mentioning is a recent initiative by several research groups from Ibero-American countries that published a compilation of carotenoid data from 191 foods, identifying 42 carotenoids [12]. Based on this carotenoid content database of Ibero-American foods, data on the phytoene and phytofluene contents in some of the foods consumed in Spain were obtained to assess the intake of these colourless carotenoids in Spain, as described in this Special Issue by Olmedilla-Alonso et al. [4]. The phytoene intake was approximately four times that of the phytofluene, which is consistent with the difference in their contents in the foods consumed by the Spanish population. Vegetables are the main source of these carotenoids, and, considering the colour of the edible part, red/orange foods account for practically all the total dietary intake. In Spain, phytoene is consumed in higher concentrations than the major carotenoids mentioned above, except for lycopene. The phytofluene intake is lower than that of the non-provitamin A carotenoids and β-carotene, but higher than the β-cryptoxanthin and α-carotene intakes.

Two papers in this Special Issue report results from a lutein-rich fruit or vegetables (three pieces/day) intervention study of normolipidemic adults consuming normal dietary amounts of these foods (500 g fruit/d or 180 g vegetables/d, which supplied 1.8 mg lutein/day) over a period of four weeks. This study elicited varying responses in terms of status biomarkers, while the serum lutein concentration increased by 37% (also consistent with its presence in faeces) and the macular pigment optical density did not exhibit any variation, nor did it correlate with the contrast sensitivity [7]. The statuses and dietary intakes of the phytoene and phytofluene in this intervention study were reported by Rodríguez-Rodríguez et al. [8], showing no significant variations in the intake or serum concentrations of these colourless carotenoids. The phytoene and phytofluene serum concentration data on 100 healthy Spanish normolipemic subjects were also furnished. Phytoene exhibited higher concentrations than phytofluene in serum, faeces, and dietary intake in healthy adults, compared to the major carotenoids studied so far in the context of diet and health. This fact highlights the need for further research on the function of these colourless carotenoids in humans, as they could also help promote health [8].

This review of the current research trends looking into the correlation between carotenoids and human health is presented in the form of an overview of the registered clinical trials (ClinicalTrials.gov, period 2000–2021), mainly covering studies on foods and food supplements, but also a combination of both sources [6]. Among the foods tested, tomatoes and tomato-based foods and eggs were the most prevalent. As for the carotenoids, trials focused mainly on lutein, lycopene, and astaxanthin. The purpose of these trials was to identify the carotenoid bioavailability and their effects on human health, mostly eye and cardiovascular health, but more recently, there has been a focus on cognitive functions and carotenoid–gut microbiota interactions. The heterogeneity of these studies points to the need for further research focusing on lesser known markers and new research areas, including the study of interactions, as well as other aspects, in well-controlled human intervention studies. 

The studies featured in this Special Issue contribute: to shedding light on long-ignored colourless carotenoids; to expanding our knowledge of lutein, a carotenoid of public health interest for its role in visual performance and reducing the risks of age-related macular degeneration, by studying the lutein status marker responses following a lutein-rich food intake in healthy subjects consuming amounts compatible with their habitual diet; to the proposal of a carotenoid dietary assessment method; and to identifying the current research trends regarding carotenoids and human health through a review of the registered clinical trials from the last twenty years.
